# Nonlinear Associations of the Built Environment with Cycling Frequency among Older Adults in Zhongshan, China

**DOI:** 10.3390/ijerph182010723

**Published:** 2021-10-13

**Authors:** Wenxiao Wang, Yi Zhang, Chunli Zhao, Xiaofei Liu, Xumei Chen, Chaoyang Li, Tao Wang, Jiani Wu, Lanjing Wang

**Affiliations:** 1 State Key Laboratory of Ocean Engineering, China Institute for Urban Governance, Shanghai Jiao Tong University, Shanghai 200240, China; wangwenxiao@sjtu.edu.cn (W.W.); cyljjf@sjtu.edu.cn (C.L.); wangtao127@sjtu.edu.cn (T.W.); JiaNiZi@sjtu.edu.cn (J.W.); lanjing.wang@sjtu.edu.cn (L.W.); 2 Transport & Roads, Department of Technology and Society, Faculty of Engineering, Lund University, 22100 Lund, Sweden; Chunli.Zhao@tft.lth.se; 3 Key Laboratory of Advanced Public Transportation Science, China Academy of Transportation Sciences, MOT, Beijing 100029, China; liuxf@motcats.ac.cn (X.L.); chenxm@motcats.ac.cn (X.C.)

**Keywords:** nonlinear, built environment, cycling, older adults, XGBoost, threshold effect

## Abstract

The health and welfare of older adults have raised increasing attention due to global aging. Cycling is a physical activity and mode of transportation to enhance the mobility and quality of life among older adults. Nevertheless, the planning strategies to promote cycling among older adults are underutilized. Therefore, this paper describes the nonlinear associations of the built environment with cycling frequency among older adults. The data were collected from the Zhongshan Household Travel Survey (ZHTS) in 2012. The modeling approach was the eXtreme Gradient Boosting (XGBoost) model. The findings demonstrated that nonlinear relationships exist among all the selected built environment attributes. Within specific intervals, the population density, the land-use mixture, the distance from home to the nearest bus stop, and the distance from home to CBD are positively correlated to the cycling among older adults. Additionally, an inverse “U”-shaped relationship appears in the percentage of green space land use among all land uses. Moreover, the intersection density is inversely related to the cycling frequency among older adults. These findings provide nuanced and appropriate guidance for establishing age-friendly neighborhoods.

## 1. Introduction

With the improvement of healthcare and welfare, the global population of older adults is increasing rapidly. Up until 2017, 9% of the world population (703 million) was 65 years old or above, and the ratio has been predicted to rise to 12% in 2030 and 16% in 2050 [1]. Currently, more than one-quarter of older adults live in Asia, North America, and Europe [2]. From 2020 to 2050, Asia may witness the fastest growth in the population of older adults [3]. As the world’s most populated country, China is estimated to possess around 380 million older adults by 2050 [4]. Global aging has highlighted the demand for the improvement of living quality among older adults. Active travel (i.e., walking and cycling) has been widely recognized as a significant intervention to promote health [5,6]. Cycling benefits cardiorespiratory fitness, musculoskeletal fitness, as well as bone health, and contributes to sleep quality via the physical activity performed while moving [7]. Older adults are advised to conduct physical activity over 150 min per week to reduce the risk of social isolation [8]. Cycling can be one of the recommended choices to keep fit [9]. Moreover, as an environmentally friendly activity [10], cycling also contributes to energy saving and air quality control [11,12].

The travel behaviors among older adults may vary in different regions. In some developed countries, driving is the top choice for daily travel among older adults [13]. In the United States, only 0.5% of older adults prefer cycling over other modes [14]. On the contrary, one-quarter of older adults in Finland choose cycling [15]. In China, 20.59% of older adults preferred cycling or using an e-bike [16]. To promote cycling among older adults, it is essential to detect the factors that may impact their cycling activity. Existing studies have investigated how the built environment is related to cycling among older adults. The significant built environment characteristics include cycling infrastructure design, distance to transit, destination accessibility, and safety. Most studies have assumed a linear relationship between various factors and cycling activity. The commonly used methods include Poisson regression [17], negative binomial regression [18], and multilevel logistic regression [19]. However, recent studies have displayed nonlinear relationships between the built environment and travel behavior [20,21,22,23,24,25,26]. A complex nonlinear association may also exist between the built environment and cycling activity among older adults.

This study contributes twofold. Empirically, the built environment–travel behavior research was enriched by investigating the nonlinearity and threshold effects of the built environment on the cycling frequency of older adults. The findings will facilitate planning practice to promote cycling activity among older adults. Technically, the eXtreme Gradient Boosting (XGBoost) model, a machine learning method, was employed. This modeling method seized an intricate relationship between the built environment and cycling frequency among older adults with explanatory variables. The results indicated that the XGBoost model was more effective than linear regression models in describing the nonlinearity of the built environment.

This paper consists of six sections. Section 2 reviews the literature on the influence of the built environment on older adults’ cycling activity and the built environment–travel behavior research with nonlinear methods. Section 3 and Section 4 introduce the data collection and modeling approach, respectively. Section 5 displays the results of the model. Section 6 discusses the results and concludes the study.

## 2. Literature Review

### 2.1. Built Environment and Cycling among Older Adults

Cycling is one of the most common physical activities among older adults [27,28]. Prior studies have attempted to explore the linear associations between the built environment and cycling activity among older adults in different contexts [19,29,30,31]. However, few studies have investigated the associations with a focus on older adults in Eastern Asia. The built environment variables prevailingly employed in the built environment–cycling activity research are categorized as the “five Ds”: density, design, diversity, distance to transit, and destination accessibility [32].

Research in China has observed a positive relationship of population density on cycling frequency among older adults [18]. However, a study in the Netherlands found that urban density is negatively correlated with older adults’ cycling activity [33]. Cycling infrastructure design has been highly discussed in prior studies due to its positive impacts on cycling activity among older adults [14,29,34,35,36]. Mixed land use development is attributed to the higher tendency of cycling for older adults [18,19,27,29,34]. Being adjacent and accessible to amenities (i.e., CBD and living services) is also linked to an increase in cycling frequency [29,30,37,38]. Living far from bus stops is found to foster cycling activity as cycling is an alternative mode for medium-distance trips if a transit service is absent [27,29,39]. Aesthetics, represented by green spaces and parks, is also a critical determinant of older adults’ cycling [40]. Additionally, safety concerns may be another influencing factor. Both the cyclists’ safety and the bicycle safety (safety from crime) have been proven to have significant effects on cycling frequency [19,30,41,42]. Hence, the improvement of the built environment may facilitate cycling among older adults.

### 2.2. Nonlinear Relationship of Travel and the Built Environment

Recent studies have attempted to describe the possible nonlinear relationships between the built environment and travel behavior with machine learning [43,44,45,46,47]. Machine learning contains various methods, including sigmoid regression, gradient boosting decision tree (GBDT), the generalized additive mixed model (GAMM), semiparametric model, random forest, and the artificial neural network (ANN). Sigmoid regression has been applied to formulate urban land density among 28 major cities in China [48]. Traffic air pollution has been predicted based on the ANN [49]. The semiparametric model has been established to analyze the associations between the built environment and electric-bike ownership [45]. The machine learning method has been gradually adopted recently (Table 1). GBDT has been adopted to investigate the associations between the built environment and travel-related outcomes, e.g., walking distance, walkability, and walking distance. The association between walking propensity, walking time, and vehicle ownership has been examined by random forest (RF). XGBoost has been employed to examine the relationships between the built environment and probability of active travel choice, travel mode, and bus use frequency.

The nonlinear models may appear to better interpret the complex relationships than the linear models can in some cases. Some studies have compared the outcomes between the nonlinear models and the linear ones. Random forest and the log-linear model (a linear model) have been selected to analyze the built environment effects on older adults’ walking [20]. The mean absolute error (MAE) and root-mean-square error (RMSE) have been introduced to examine two models. The outcomes showed that the values of MAE and RMSE for the linear model were higher, which means that the regression effect was worse. Two machine learning models (nonlinear models) and a conventional land-use regression model (a linear model) have been adopted to predict traffic air pollution, and the normalized root-mean-square error (NRMSE) has been introduced to evaluate the models. The result showed that the values of the NRMSE for the linear model were higher, which means that the predictions were less precise [50].

The studies mentioned above acknowledged the intricate nonlinearity of the built environment on travel and advice to acquire more efficient environmental interventions based on the threshold influence. However, the relevant investigation of the nonlinear effect of the built environment on cycling among older adults is rare and remains to be further explored.

**Table 1:** Selected studies on the relationships between the built environment and travel behavior employing machine learning algorithm.

Method	Study	Dependent Variable	Built Environment Variable
GBDT	(Huang et al., 2021) [23]	Total time of physical activity	Land-use mix, population density, Number of transit stops, Number of intersections, Greenness, Distance from home to school, Distance to the nearest park
	(Tao et al., 2020) [51]	Walking distance	Population density, Pedestrian network density, Intersection density
	(Dong et al., 2019) [52]	Walkability	Number of walking routes, Traffic safetySecurity from crime, Streetlight exposureSize of open space, Green space quality
RF	(Yang et al., 2021) [26]	Walking propensity	Population density, Land-use mix, Intersection density, Access to bus stops Access to recreational facilities, Streetscape greenery
	(Sabouri et al., 2020) [53]	Vehicle ownership	transit stop density, intersection density, percentage of 4-way intersections, population density, housing density
	(Cheng et al., 2020) [20]	Walking time	Land-use mix, Street connectivity, Number of bike-sharing stations, Number of bus stops, Distance to the nearest square/park, Distance to the nearest card/chess room
XGBoost	(Liu et al., 2021) [24]	Probability of choosing active travel divided by the probability of not choosing active travel	Population density, Job density, Land-use mix, Intersection density, Bus stop density, Distance to city centre
	(Kim, 2021) [54]	Travel mode	Land use, Number of subway stops, Population density, Number of bus stops
	(Wang et al., 2021) [55]	Bus use frequency	Dwelling units’ density, Intersection density, Land-use mixture, Area coverage of commercial establishments within 1 km from the center of a neighborhood, Bus-stop density, Percentage of green space land use among all land uses

## 3. Data

### 3.1. Study Case

**Figure 1:**
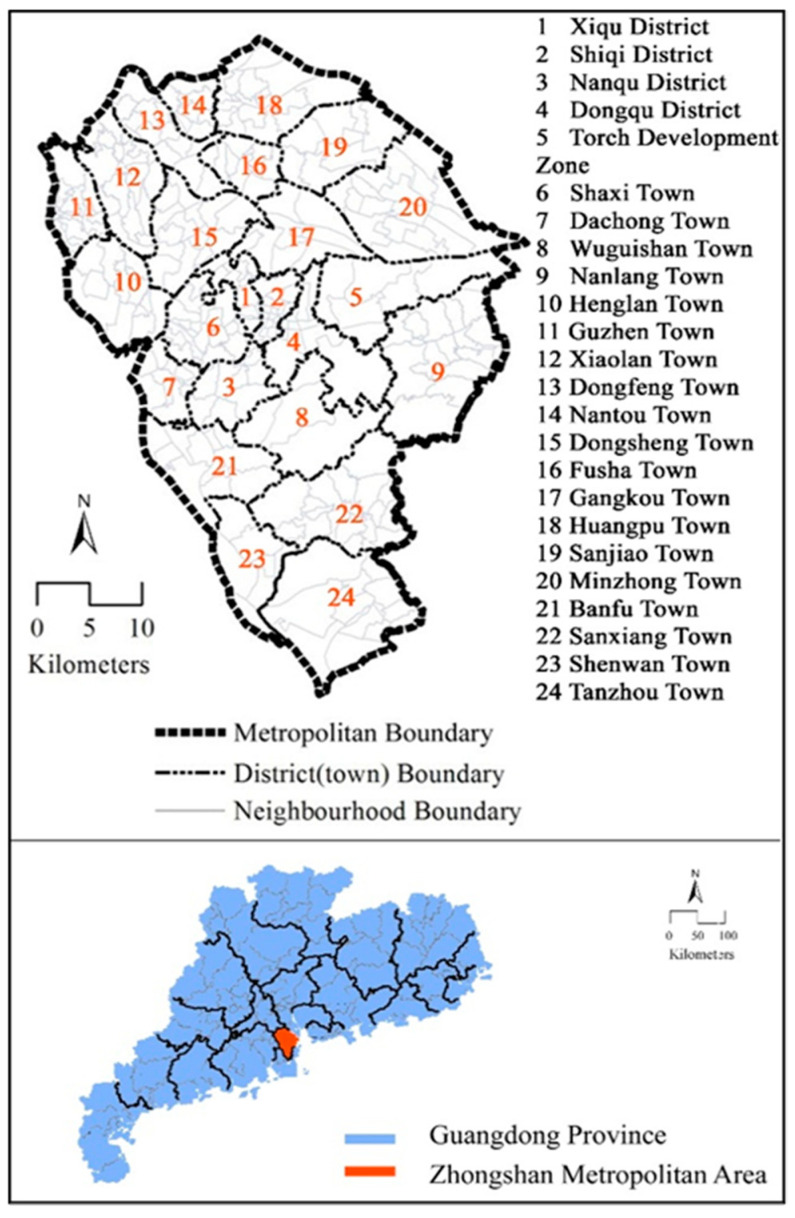
Study area.

The study case was Zhongshan City in Guangdong Province, China (Figure 1). Zhongshan is located in the Guangdong–Hong Kong–Macao Greater Bay Area, one of the most economically developed city clusters in China. In those city clusters, there are about 20 cities with similar urbanization and motorization levels and urban transportation characteristics to Zhongshan [29]. Therefore, the findings in Zhongshan may also be informative to similar cities.

Selected by stratified random sampling covering the whole Zhongshan Metropolitan Area, the ZHTS 2012 provided the self-reported one-day cycling activity, e.g., frequency, duration, purpose of cycling trips, together with the personal and household data of respondents. The survey was stratified by the 274 neighborhoods. In each neighborhood, the sample size was determined by the population of the neighborhood. The sample size of older adults in Zhongshan was 4784 (2905 male and 1879 female) from 274 urban and rural neighborhoods, with a sample rate of 2%. Among the respondents, 777 (16.2%) cycled at least one time per day.

### 3.2. Characterization of Built Environment Attributes

The built environment variables were defined based on neighborhoods [56]. In Zhongshan, a neighborhood is homogeneous in terms of socio-demographics and living conditions [57]. According to the administrative division of Zhongshan, the entire 274 neighborhoods were selected in this study. These neighborhoods cover 1783.67 km^2^. The average size of a neighborhood is 6.51 km^2^. The following data for the characterization of built environment attributes came from the Zhongshan Municipal Bureau of Urban Planning: (1) neighborhood boundaries; (2) land use in 2012 with five major types of land use (residential land, commercial and service facilities, industrial and manufacturing, green space, and other types of land uses); (3) population, dwelling units, and employment in 2012; (4) road networks; (5) bus stops; and (6) political boundaries such as city and zone boundaries. All the data were then integrated into ArcGIS for further analysis.

In a built environment–travel behavior review [32], the built environment characteristics were divided into five categories. They were defined as “5Ds” variables as the five categories start with “D,” including density, design, distance to transit, destination accessibility, and diversity. Based on the best available data, this study selected one representative variable from each of the 5Ds (Table 2). Therefore, the five variables chosen were population density (density), intersection density (design), distance from home to the nearest bus stop (distance to transit), distance from home to CBD (destination accessibility), and land-use mixture (diversity). The percentage of green space land use among all land uses signifies the aesthetics of the urban environment. Therefore, we chose this variable as the sixth built environment variable (Table 3).

The population density, intersection density, distance to the nearest bus stop, distance from home to CBD, and the percentage of green space land use are self-explanatory. Land-use mixture refers to the degree of mixing of different land uses in the neighborhood, usually characterized by the entropy index (EI) [58] as follows:

(1) $$EI=\overset{n}{\underset{i=1}{\sum }}P_{i}\log\left(1/P_{i}\right)$$

where *n* represents the number of different functions of land; $P_{i}$ represents the percentage of land use i’s land coverage over total land coverage. Among them, 0 represents completely single land use, and 1 represents equalized land use for different purposes in the selected area.

## 4. Method

The XGBoost Model is a machine learning method proposed by Dr. Tianqi Chen in 2016 [59]. XGBoost has many advantages over traditional linear regression models. First, XGBoost can characterize the nonlinear relationship between the independent and dependent variables. Therefore, no assumption that a specific relationship between the independent variable and the dependent variable is acquired when using the XGBoost model. Secondly, the XGBoost model introduces a regular term, which can effectively prevent the model from overfitting. Additionally, XGBoost takes the second derivative of the approximation term when performing Taylor expansion approximation on the objective function. Accordingly, the calculation loss is smaller compared to the traditional decision tree models (i.e., gradient boosting decision tree, GBDT). Meanwhile, the process of calculation is simplified and the operating efficiency is improved as column sampling is supported by XGBoost. However, XGBoost, a machine learning model, cannot provide thorough information for statistical inference compared with statistical models. The algorithm process of XGBoost is as follows.

Step 1: Construct the objective function, see Formula (2).

(2) $$obj=\overset{n}{\underset{i=1}{\sum }}l\left(y_{i},{\hat{y}}_{i}\right)+\underset{k=1}{\sum }\Omega \left(f_{k}\right)$$

where ${\hat{y}}_{i}=\overset{K}{\underset{k=1}{\sum }}f_{k}{\left(x_{i}\right)}_{,}f_{k}\epsilon F$, K is the number of trees, $F=\left\{f\left(x\right)=w_{q\left(x\right)}\right\}\left(q:{\mathbb{R}}^{m}\to T,\;w\in {\mathbb{R}}^{T}\right)$, T is the number of nodes, w is the weight of the node, and $\Omega \left(f_{k}\right)$ is the regular term.

Step 2: Optimize the objective function.

Transfer Formula (2) into Formula (3),

(3) $$\min\;\;obj=\overset{n}{\underset{i=1}{\sum }}l\left(y_{i},{\hat{y}}_{i}{}^{\left(k-1\right)}+f_{k}\left(x_{i}\right)\right)+\Omega \left(f_{k}\right)$$

and expand Formula (3) using Taylor series to acquire Formula (4),

(4) $$\min\;\;obj=\overset{n}{\underset{i=1}{\sum }}[g_{i}\cdot f_{k}\left(x_{i}\right)+\frac{1}{2}h_{i}\cdot f_{k}^{2}\left(x_{i}\right)]+\Omega \left(f_{k}\right)$$

where $g_{i}=\frac{\partial l\left(y_{i},{\hat{y}}_{i}{}^{\left(k-1\right)}\right)}{\partial {\hat{y}}_{i}{}^{\left(k-1\right)}}$ and $h_{i}=\frac{\partial^{2}l\left(y_{i},{\hat{y}}_{i}{}^{\left(k-1\right)}\right)}{\partial^{2}{\hat{y}}_{i}{}^{\left(k-1\right)}}$.

Step 3: Introduce the structure of the tree into the objective function.

Replace the regular term with Formula (5),

(5) $$\Omega \left(f_{k}\right)=\gamma T+\frac{1}{2}\lambda \sum_{j=1}^{T}\omega_{j}^{2}$$

where γ is the threshold parameter and λ is the regularization parameter.

Incorporate Formula (5) into Formula (4) and simplify it to acquire Formula (6).

(6) $$\min\;\;obj=\overset{T}{\underset{j=1}{\sum }}[G_{j}\cdot \omega_{j}+\frac{1}{2}\left(H_{i}+\lambda \right)\cdot \omega_{j}^{2}]+\gamma T$$

The optimal objective function is obtained by deriving $\omega_{j}$ in Formula (6), see Formula (7) for details.

(7) $$obj^{*}=-\frac{1}{2}\overset{T}{\underset{j=1}{\sum }}\frac{G_{j}{}^{2}}{H_{j}+\lambda }+\gamma T$$

Step 4: Determine the structure of the tree.

According to Formula (7), calculate the difference between the loss function value of the node before and after the split as the characteristic value, see Formula (8) for details.

(8) $$\max\;obj_{old}^{*}-obj_{new}^{*}=\frac{1}{2}\left[\frac{G_{L}^{2}}{H_{L}+\lambda }+\frac{G_{R}^{2}}{H_{R}+\lambda }-\frac{{\left(G_{L}+G_{R}\right)}^{2}}{{\left(H_{L}+H_{R}+\lambda \right)}^{2}}\right]-\gamma$$

In this paper, the greedy algorithm is applied to solve Formula (8).

## 5. Results and Discussion

Before modeling the XGBoost, a variance inflation factor (VIF) test was performed to examine the possible multicollinearity among independent variables. The VIF values of independent variables displayed in Table 4 were less than 10, a threshold for excluding variables [60]. Then, we applied the XGBoost approach to distinguish the relative importance of selected variables and to illustrate the nonlinear association with the built environment variables. We used the “xgboost” package in Python to establish the XGBoost model. The parameters were all default ones. Finally, we compared the prediction accuracy among XGBoost, GBDT, and multilinear regression.

### 5.1. Relative Importance of Independent Variables

**Figure 2:**
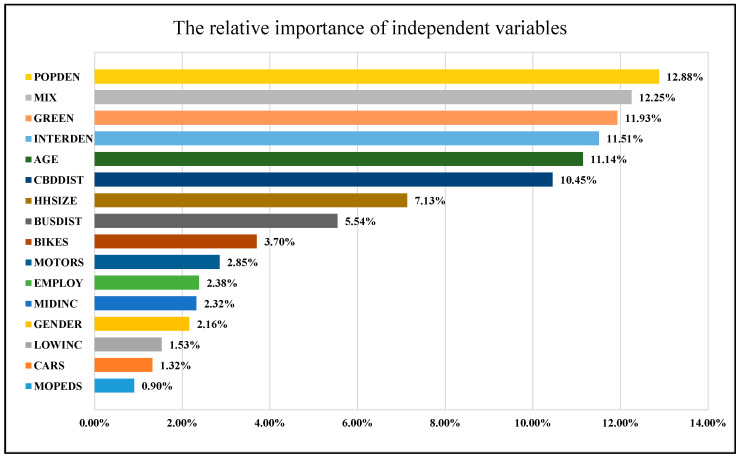
The ranking of the relative importance of independent variables.

Table 5 shows the relative importance of the selected independent variables. When the relative importance ratio is higher, the corresponding independent variables are more significant to the dependent variable (Figure 2). The relative importance ratios of the built environment, household characteristics, and individual characteristics are 64.57%, 19.75%, and 15.68%, respectively. The built environment has a greater impact on the cycling frequency among older adults than household or individual characteristics do. Particularly, in certain thresholds, the population density, land-use mixture, percentage of green space land use among all land uses, intersection density, and distance to CBD will promote cycling among older adults as their relative importance is above the average (6.25%). The outcome reinforces the findings in the existing literature that the built environment exerts more influence on travel behavior than sociodemographics do [32].

### 5.2. Nonlinear Associations of the Built Environment Variables

**Figure 3:**
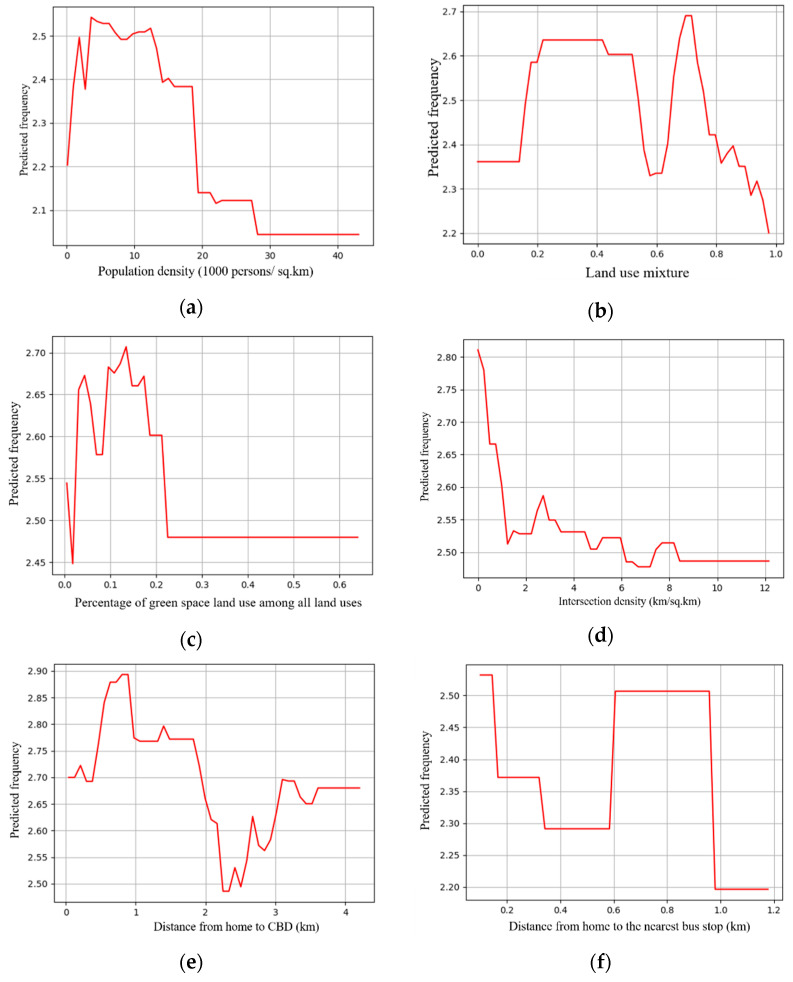
(**a**) Association between population density and frequency; (**b**) association between land-use mixture and frequency; (**c**) association between percentage of green space land use among all land uses and frequency; (**d**) association between intersection density and frequency; (**e**) association between distance from home to CBD and frequency; (**f**) association between distance from home to the nearest bus stop and frequency.

Previous studies have assumed that there was a linear or log-linear relationship between the built environment and travel behaviors. However, this assumption sometimes failed to reflect the complex relationship between the two [61]. In this section, we explored the nonlinear relationships between the built environment and cycling frequency among older adults by employing the XGBoost model. As a means of extracting the influence of the single built environment variable, the partial dependence plot (PDP) (Figure 3) can visualize the marginal effects of the independent variables on the dependent variables [62]. In Figure 3, the X-axes represent the six built environment variables, and the Y-axes represent the predicted cycling frequency among older adults.

**Figure 4:**
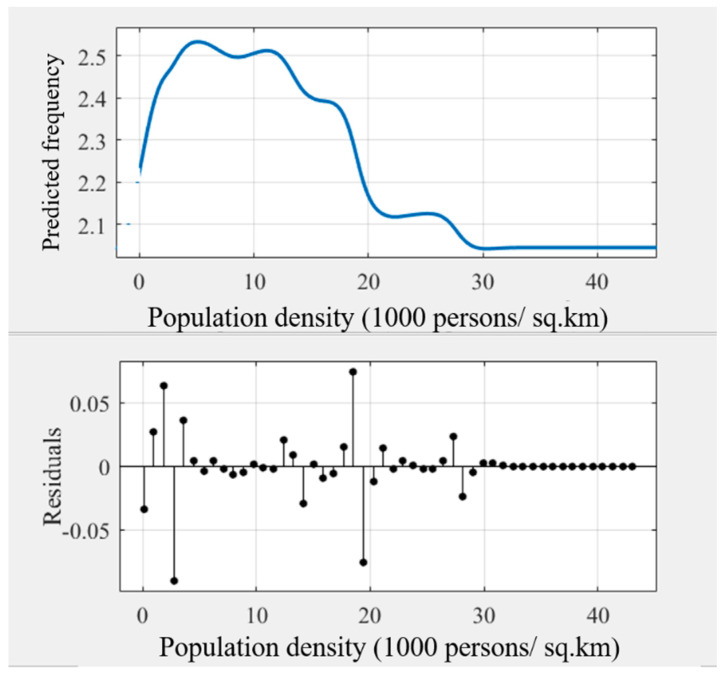
Nonlinear associations between POPDEN (population density) and cycling frequency among older adults.

**Figure 5:**
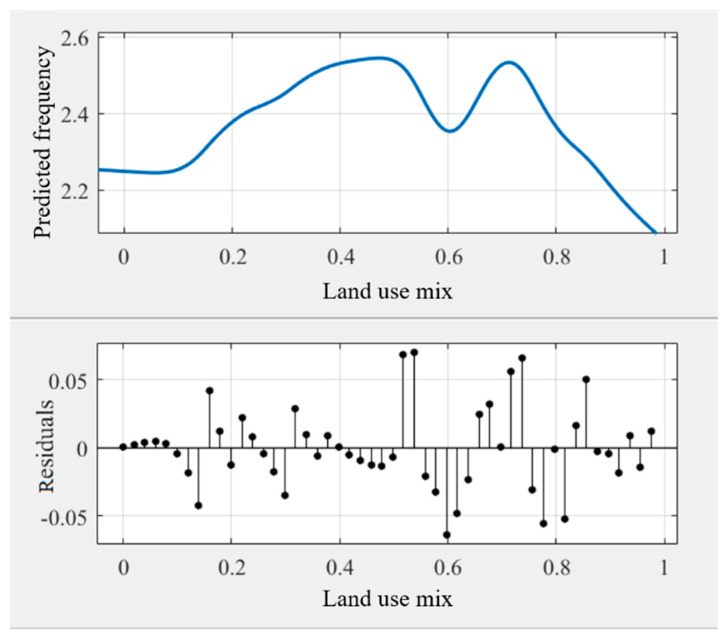
Nonlinear associations between land-use mixture (MIX) and cycling frequency among older adults.

**Figure 6:**
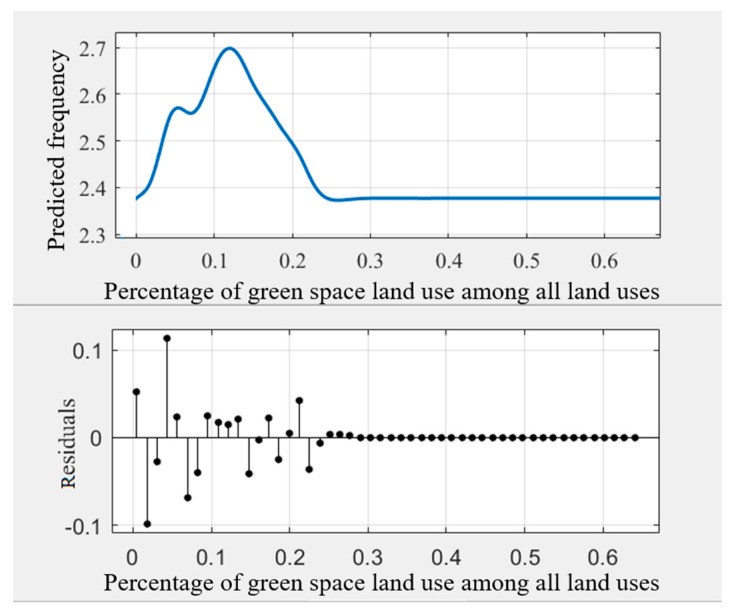
Nonlinear associations between the percentage of green space land use among all land uses (GREEN) and cycling frequency among older adults.

**Figure 7:**
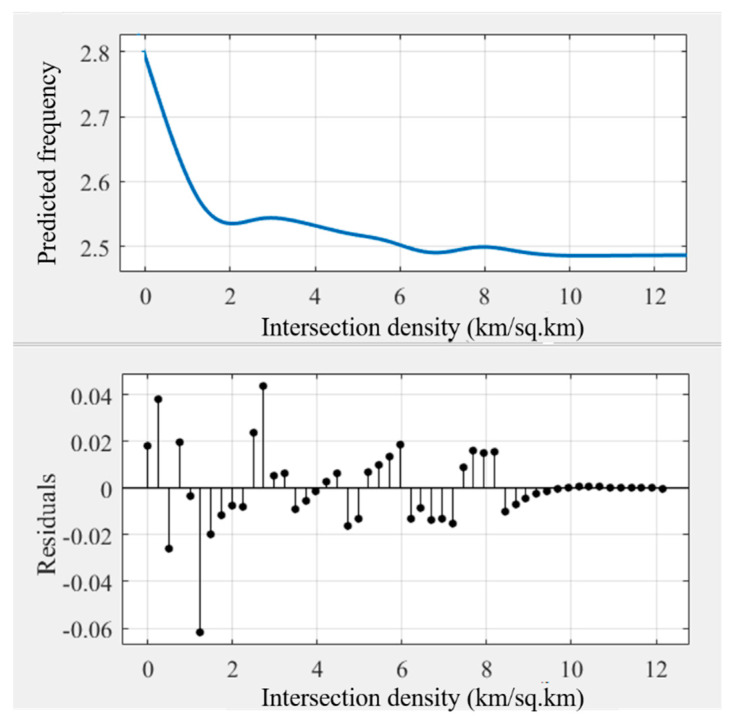
Nonlinear associations between intersection density (INTERDEN) and cycling frequency among older adults.

**Figure 8:**
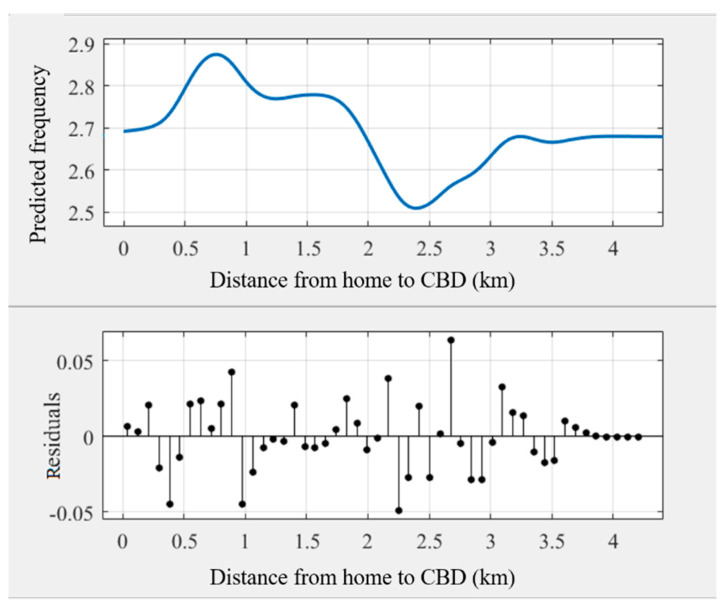
Nonlinear associations between the distance from home to the CBD (CBDDIST) and cycling frequency among older adults.

**Figure 9:**
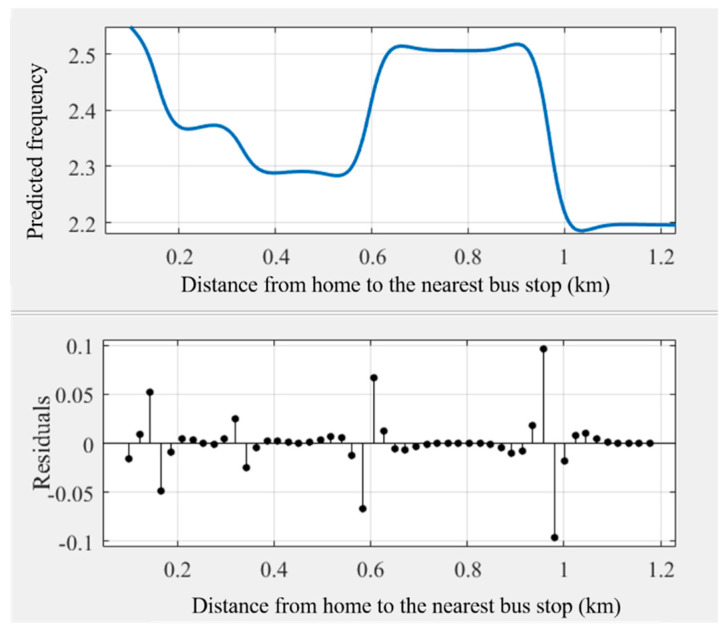
Nonlinear associations between the distance from home to the nearest bus stop (BUSDIST) and cycling frequency among older adults.

As some noise occurred in the modeling results (Figure 3), the general trend of the relationships may be hindered. Some prior studies transferred the original curves into smoothing curves to obtain intuitive relationships [45,51]. This study used Matlab’s cftool toolbox to obtain the smoothing curves (Figure 4, Figure 5, Figure 6, Figure 7, Figure 8 and Figure 9). As illustrated in Figure 4, Figure 5, Figure 6, Figure 7, Figure 8 and Figure 9, all the six built environment variables have relatively complex nonlinear associations with cycling frequency among older adults. We discuss the results of the six built environment variables in order of relative importance.

#### 5.2.1. Nonlinear Associations of Population Density

For population density, the cycling frequency peaks at 5000 persons per square kilometer after a rapid surge. Then, it drops with a fierce fluctuation after 5000 persons per km^2^. Finally, the curve becomes flat beyond 30,000 persons per km^2^. The results imply that the population density of 5000 persons/km^2^ is sufficient to promote cycling among old adults. That echoes the results of a recent study in Zhongshan on the nonlinearity of the built environment on walking [25]. When the population density is around 5000 persons/km^2^, the thresholds appear both in walking and cycling frequency among older adults. In ultra-densely populated areas, active travel (e.g., walking and cycling) among older adults is negatively related to population density. This finding is also consistent with Cerin et al.’s work that additional population in highly compact neighborhoods may even reduce the propensity for active travel among older adults [63].

#### 5.2.2. Nonlinear Associations of Land-Use Mixture

The cycling frequency among older adults is associated with the land-use mixture in an M shape. After a steady climb, the cycling frequency arrives at its first peak at around 0.5 (entropy index). After an approximate “V”-shaped fluctuation bottoming at about 0.6, it then reaches the second peak at 0.7, preceding a rapid drop within the range of 0.7 to 1.0. When the land use is around 0.5 and 0.7, the thresholds occur, while the threshold of walking appears when the land-use index is 0.7 [25]. The results imply that highly mixed land use may reduce the likelihood of older adults choosing cycling, consistent with recent studies in Eastern-Asian cities (e.g., Seoul and Hong Kong) [64,65]. It is reasonable that older adults are prone to forming a chain of multiple trips in one journey if residing closer to services and destinations [66,67]. Nevertheless, further research is needed to reveal the in-depth reasons.

#### 5.2.3. Nonlinear Associations of the Percentage of Green Land Use among All Land Uses

For the percentage of green land use among all land uses, when it falls within 25%, the curve shows an approximate inverse V shape with a peak at 12%. Then, the influences become trivial after 25%. The results indicate that the percentage of green land use among all land uses is most effective from 0% to 12%. Within this range, abundant street trees and green corridors provide a cycling-friendly environment, contributing to the increased cycling trips among older adults. When the percentage of green space land use is beyond 25%, the older adults tend to cycle less, in line with prior studies [68]. Presumably, in neighborhoods with a higher proportion of green land use, the commercial and service establishments are sparsely distributed and beyond the suitable cycling distance for older adults. The threshold of cycling occurs when the GREEN is around 0.13, while that for walking occurs when the GREEN is around 0.4 [25].

#### 5.2.4. Nonlinear Associations of Intersection Density

Generally, the intersection density is negatively correlated with the cycling frequency among older adults. Within the range of 0 to 2.0 intersections per km^2^, the cycling frequency undergoes a sharp decrease in a nearly linear pattern. Afterward, it lessens steadily with a mild fluctuation until 12 km per km^2^. The association indicates that in neighborhoods with more intersections, the propensity of older adults to cycle is lower. Oftentimes, denser intersections in Zhongshan imply a higher volume of traffic mixed with cars, motorcycles, e-bikes, bikes, and pedestrians. Similar to our findings, prior studies have demonstrated that, in China, the risk of traffic accidents for cyclists is high at intersections [69]. Due to safety concerns, older adults may decide to opt for modes other than cycling [6].

#### 5.2.5. Nonlinear Associations of the Distance to the CBD

As for the distance to the CBD, the nonlinearity pattern is intricate. Generally, a flat “s”-shaped curve occurs. The cycling frequency climbs steadily before peaking at 0.8 km. Then, it fluctuates downward before a “V”-shaped curve appears in the range of 1.7 to 3.2 km. Afterward, the curve becomes flat. As a low-speed travel mode, cycling can be time- and strength-consuming for long-distance trips. Therefore, it is consistent with our expectations that a negative association occurs when the distance to the CBD is from 0.8 km to 2.4 km. However, when the distance to the CBD is beyond 2.4 km, the cycling frequency among older adults is positively correlated. This is reasonable because the urban structure of Zhongshan is polycentric. The subcenters are located over 2 km away from the CBD, and hence, older adults may travel to the subcenters for daily activities.

#### 5.2.6. Nonlinear Associations of the Distance from Home to the Nearest Bus Stop

The cycling frequency drops stably when the distance from home to the nearest bus stop increases from 0.1 to 0.5 km. Afterward, an approximate inverted “U”-shaped fluctuation occurs, peaking at about 0.65 to 0.9 km. Following a sharp dive, the cycling frequency remains flat beyond 1 km afterward. Oftentimes, when the nearest bus stop is located within 0.5 km, older adults would opt for walking to bus stops. However, if the distance is beyond a suitable walking distance, which is from 0.5 to 0.95 km in Zhongshan, they may change to cycling over walking. That may explain the threshold effect of the distance from home to the nearest bus stop on cycling frequency among older adults.

### 5.3. Model Comparison with Linear Regression

We also applied a conventional multiple linear regression model and a GBDT model in this study for comparison with the XGBoost model. Table 6 presents the results of the multiple linear regression.

The mean square error (MSE), mean absolute error (MAE), and mean absolute percentage error (MAPE) of the three models were calculated to test the preciseness of prediction. The model performs better when these metric values are as small as possible. These metrics are formulated as follows.

(9) $$MSE=\frac{1}{N}\overset{N}{\underset{i\;=1}{\sum }}{\left(\hat{y_{i}\;}-\;y_{i}\right)}^{2}$$

(10) $$MAE=\;\frac{1}{n}\overset{n}{\underset{i=1}{\sum }}\left|{\hat{y}}_{i}-y_{i}\right|$$

(11) $$MAPE=\;\frac{100\%}{n}\overset{n}{\underset{i=1}{\sum }}\left|\frac{{\hat{y}}_{i}-y_{i}}{y_{i}}\right|$$

where *N* is the total number of samples, $\hat{y_{i}\;}$ is the predicted value for the $i^{th}$ sample, and $y_{i}$ is the observed value for the $i^{th}$ sample.

The metric values of the three models are shown in Table 7. The XGBoost model performs best in prediction among the three models.

## 6. Conclusions

This study investigated the nonlinear associations between the built environment and cycling frequency among older adults based on the XGBoost model. The dedication of the research is summarized into three points.

First, the outcomes showed that the hypothesis of nonlinear associations between the built environment and cycling frequency among older adults is valid. According to the results, the nonlinearity is presented in all the six built environment characteristics. A model comparison was also conducted in the perspective of prediction precision among multilinear regression, GBDT, and XGBoost. The result demonstrated that XGBoost is more accurate in the prediction of cycling frequency based on the selected built and socioeconomic attributes. Accordingly, the nonlinear methods are more suitable for cycling frequency prediction.

Second, the results highlighted the critical roles of built environment characteristics in influencing the cycling frequency among older adults. Within certain ranges, all else unchanged, denser population, mixed land-use development, fewer intersections, more convenient bus service, and abundant green space land use may arouse older adults’ desire to cycle. Although the conclusions may not be directly transferrable in other areas, the modeling approach in this paper is applicable in other contexts to facilitate strategies for land use and transport planning.

Third, the results indicated that the built environment characteristics have obvious threshold effects on cycling frequency among older adults. A single built environment attribute may have inequivalent effects across the whole range of that attribute. Hence, discovering the proper interval may be economical. In Zhongshan, the population density of around 5000 persons/km^2^ may be appropriate for increasing cycling frequency among older adults. Additionally, to promote cycling among older adults, land-use mixture entropy indexes of 0.5 and 0.7 are advisable. Moreover, for the percentage of green space land use among all land uses, the suggested value for encouraging older adults to cycle is around 12%.

This study has some limitations. First, the confidence interval of the predicted value cannot be calculated based on the XGBoost model. Presumably, the distributions of variables are difficult to obtain. Accordingly, the pivot quantity cannot be established. Secondly, due to the data availability, this study did not include all the variables that are relatively important to the cycling frequency among older adults. In future studies, other variables will be incorporated. Thirdly, this research was based on cross-sectional data. As with some of the prior studies, the current work was unable to clearly verify the causal effects of the built environment on cycling frequency among older adults.

## Figures and Tables

**Table 2:** The description of built environment variables.

Category	Meaning	The Built Environment Variables Used in This Study
Density	The variables of interest per unit of area	Population density (POPDEN)
Design	The characteristics of the street network inside an area	Intersection density (INTERDEN)
Distance to transit	The level of transit service at the residences or workplaces	Distance from home to the nearest bus stop (BUSDIST)
Destination accessibility	Ease of access to a certain location	Distance from home to CBD (CBDDIST)
Diversity	The number of different land uses in a fixed area and the represented degree	Land-use mixture (MIX)
Aesthetics	Attractiveness and appeal of a place	Percentage of green space land use among all land uses (GREEN)

**Table 3:** The description of variables used in the analysis.

Variable	Description	Mean/Percentage(%)	SD
Cycling Activity			
Frequency	Daily cycling frequency	2.31	0.82
Household Characteristics			
HHSIZE	The population of a household, count	2.76	1.42
HIGHINC	High annual household income (over RMB 150,000), binary, 1 = yes, 0 = no	13	\
MIDINC	Medium annual household income (RMB 50,000~150,000), binary, 1 = yes, 0 = no	48	\
LOWINC	Low annual household income (RMB 50,000~150,000), binary; 1 = yes, 0 = no	39	\
BIKES	Bicycle ownership in a household, count	1.18	0.63
MOPEDS	E-bike ownership in a household, count	0.19	0.43
MOTORS	Motorcycle ownership in a household, count	0.76	0.84
CARS	Car ownership in a household, count	0.17	0.46
Individual Characteristics			
GENDER	Male	71	\
	Female	29	\
AGE	Age of respondents (≥60)	65.59	5.69
EMPLOY	Employed	35	\
	Retireed	65	\
Built Environment			
POPDEN	Population density, 1000 persons/km^2^; continuous	5.44	7.47
INTERDEN	Intersection density, km/km^2^, continuouse	2.05	2.61
BUSDIST	Distance from home to the nearest bus stop, km, continuous	0.55	0.38
CBDDIST	Distance from home to CBD, km, continuous	1.89	1.05
MIX	Land-use mixture, entropy index, continuous	0.72	0.18
GREEN	Percentage of green space land use among all land uses, continuous	0.06	0.07

Note: SD = standard deviation.

**Table 4:** The results of VIF test.

Variable	VIF
Household Individual characteristics	
HHSIZE (Household population)	3.375
MIDINC (Medium-income household)	3.876
LOWINC (Low-income annual household)	5.869
BIKES (Bicycle ownership)	1.416
MOPEDS (E-bike ownership)	1.558
MOTORS (Motorcycle ownership)	5.503
CARS (Car ownership)	1.693
Individual characteristics	
GENDER	2.160
AGE	1.520
EMPLOY (Employment status)	1.757
Built Environment	
POPDEN (Population density)	6.931
INTERDEN (Intersection density)	5.135
BUSDIST (Distance from home to the nearest bus stop)	1.490
CBDDIS (Distance from home to CBD)	1.544
MIX (Land-use mixture)	1.378
GREEN (Percentage of green space land use among all land uses)	1.634

**Table 5:** The relative importance of independent variables.

Variable	Relative Importance (%)	Rank	Total (%)
Household Characteristics			
HHSIZE	7.13	7	19.75
MIDINC	2.32	12	
LOWINC	1.53	14	
BIKES	3.70	9	
MOPEDS	0.90	16	
MOTORS	2.85	10	
CARS	1.32	15	
Individual Characteristics			
GENDER	2.16	13	15.68
AGE	11.14	5	
EMPLOY	2.38	11	
Built Environment			
POPDEN	12.88	1	64.57
INTERDEN	11.51	4	
BUSDIST	5.54	8	
CBDDIST	10.45	6	
MIX	12.25	2	
GREEN	11.93	3	
Total relative importance			100

**Table 6:** The details of multilinear regression.

Variable	Coefficient	T-Statistic	*p*-Statistic
Household Characteristics			
HHSIZE	0.08	0.354	0.726
MIDINC	−0.342	−1.406	0.169
LOWINC	−0.252	−0.842	0.406
BIKES	−0.145	−0.983	0.333
MOPEDS	−0.126	−0.817	0.42
MOTORS	−0.012	−0.043	0.966
CARS	0.377	2.34	0.025 *
Individual Characteristics			
GENDER	0.002	0.012	0.99
AGE	−0.071	−0.467	0.644
EMPLOY	0.169	1.032	0.31
Built Environment Characteristics			
POPDEN	−0.45	−1.382	0.176
INTERDEN	0.383	1.366	0.181
BUSDIST	−0.068	−0.45	0.656
CBDDIST	0.26	1.69	0.101
MIX	−0.077	−0.534	0.597
GREEN	0.119	0.751	0.458

Note: * *p* < 0.05.

**Table 7:** The metric values of the three models.

	Metric	MSE	MAE	MAPE
Model				
Multilinear regression		0.614	0.564	25.581
GBDT		0.589	0.539	24.356
XGBoost		0.585	0.503	22.429

## Data Availability

The dataset presented in this article are not readily available, because it belongs to the Zhongshan Municipality Natural Resources and Planning Bureau and is a part of the ongoing projects (Grant No. 18BSH143 of the National Social Science Foundation of China, Grant No. 20692109900, and Grant No. 21692106700 of Shanghai Science and Technology Program, and Grant No. 2020-APTS-04 of APTSLAB). Therefore, the dataset is confidential during this period.
